# Population variation alters aggression-associated oxytocin and vasopressin expressions in brains of Brandt’s voles in field conditions

**DOI:** 10.1186/s12983-021-00441-w

**Published:** 2021-10-30

**Authors:** Shuli Huang, Guoliang Li, Yongliang Pan, Jing Liu, Jidong Zhao, Xin Zhang, Wei Lu, Xinrong Wan, Charles J. Krebs, Zuoxin Wang, Wenxuan Han, Zhibin Zhang

**Affiliations:** 1 State Key Laboratory of Integrated Pest Management, Institute of Zoology, Chinese Academy of Sciences, Beijing, 100101 China; 2 CAS Center for Excellence in Biotic Interactions, University of Chinese Academy of Sciences, Beijing, 100049 China; 3 School of Medicine, Huzhou University, Huzhou, 313000 China; 4 Department of Zoology, University of British Columbia, Vancouver, BC V6T 1Z4 Canada; 5 Department of Psychology and Program in Neuroscience, Florida State University, Tallahassee, FL 32306-1270 USA; 6 College of Resources and Environmental Sciences, China Agricultural University, Beijing, 100193 China

**Keywords:** Social stress, Aggression behavior, Oxytocin (OT), Vasopressin (AVP), Density-dependency, Population regulation, Rodent

## Abstract

Density-dependent change in aggressive behavior contributes to the population regulation of many small rodents, but the underlying neurological mechanisms have not been examined in field conditions. We hypothesized that crowding stress and aggression-associated oxytocin (OT) and arginine vasopressin (AVP) in specific regions of the brain may be closely related to aggressive behaviors and population changes of small rodents. We analyzed the association of OT and AVP expression, aggressive behavior, and population density of Brandt’s voles in 24 large semi-natural enclosures (0.48 ha each) in Inner Mongolia grassland. We tested the effects of population density on the OT/AVP system and aggressive behavior by experimentally manipulating populations of Brandt’s voles in the grassland enclosures. High density was positively and significantly associated with more aggressive behavior, and increased expression of mRNA and protein of AVP and its receptor, but decreased expression of mRNA and protein of OT and its receptor in specific brain regions of the voles. Our study suggests that changes in OT/AVP expression are likely a result of the increased psychosocial stress that these voles experience during overcrowding, and thus the OT/AVP system can be used as indicators of density-dependent stressors in Brandt’s voles.

**Supplementary Information:**

The online version contains supplementary material available at 10.1186/s12983-021-00441-w.

## Background

Understanding the mechanisms of population regulation in small mammals has long been a fundamental topic in population ecology [1, 2]. Hypotheses addressing population fluctuations and regulation in small rodents are generally classified into two categories: extrinsic or intrinsic hypotheses. Extrinsic hypotheses emphasize the role of climate [3], predators [4] and food [5] in causing population fluctuations, while intrinsic hypotheses emphasize the role of density-dependent genetics [6], physiology [7] and behavior [8, 9]. Both extrinsic and intrinsic factors can jointly contribute to population regulation in small mammals.

Density-dependence is well recognized in studies of population regulation driven by various intrinsic factors [10, 11]. The genetic regulation hypothesis [6], the physiological regulation hypothesis [7, 12] and the social behavioral regulation hypothesis [8] are widely used to explain density-dependence in populations of small rodents. The genetic hypothesis suggests that population density exerts selective pressure on different genotypes, favoring highly aggressive and low reproductive animals in high density, and vice versa. The behavioral regulation hypothesis suggests that territory defense, social rank and aggressive behavior play key roles in regulating populations; animals with low aggressive behavior or social rank suffer low reproduction and high mortality due to lack of resources [8]. The physiological regulation hypothesis posits that high population density induces high aggression and high social stress, which result in impairment of the hypothalamus–pituitary–adrenal (HPA) axis and the hypothalamic–pituitary–gonadal (HPG) axis including increases in corticosterone (CORT), decreases in growth or reproductive hormones, followed by a population crash or decline [7, 13]. It is notable that all the three core hypotheses include the role of density-dependent aggressive behavior in regulating population fluctuations. Under laboratory conditions, high density induced aggression or fighting alters oxytocin (OT) and arginine-vasopressin (AVP) expression in brains of Brandt’s voles [14]. But how population density affects aggressive behavior (and then population growth) of animals via neurobiological pathways has not been investigated in field conditions.

Animals face with various environmental stressors in field conditions, including food shortage, crowding and aggression. When individuals are under stress, various input information gathers in the paraventricular nucleus (PVN) of the hypothalamus which synthesizes AVP and activates corticotropin releasing hormone (CRH), leading to the activation of adrenocorticotrophin (ACTH). ACTH enters the blood, acts on the adrenal cortex, and promotes the release of glucocorticoids (cortisol in humans, CORT in rodents; hereafter GCs) which acts as negative feedback on the HPA stress axis [15–17]. However, if the stress process is prolonged, this leads to chronic stress which affects the central nervous system (CNS). Changes in neurobiochemistry have a negative impact on the function of the HPA axis, and thus cause organic damage to the brain [18, 19]. Experiments in laboratory rats have shown that depression and anxiety are all related to chronic social stress [20]. Meynen et al. reported that the expression levels of AVP in the supraoptic nucleus (SON) and PVN in patients with melancholic depression were significantly higher than those in normal controls [21]. The high levels of CRH and GCs that are secreted under stress can inhibit the secretion of hypothalamic gonadotropin-releasing hormone (GnRH), reduce the secretion of luteinizing hormone (LH) and follicle-stimulating hormone (FSH), and regulate the reproductive ability of animals [22, 23]. OT also shows inhibitory effects on the HPA axis in waxbills and rats [24, 25].

Aggressive behavior is important for survival of many animals. Animals may adopt an "offensive" model as a physiological strategy when coping with social stress [26–28]. Studies on the relationship between OT and AVP and aggressive behavior have shown that OT and AVP mediate selective aggression and the formation of affiliation [25, 29]. OT can inhibit the activity level of medial amygdala (MeA), reduce the release of ACTH and GCs, and reduce the individual's anxiety level, thereby reducing aggressive behavior [30]. High circulating GCs concentration under high stress conditions will increase aggressive behavior which can also promote the release of male hormones (i.e., testosterone, dehydroepiandrosterone) [31, 32].

Social stress can change the expression of AVP and OT and their release [33], and thus regulate the aggressive behavior of rodents. In general, releasing of hormones involved in the stress-response, such as AVP could promote the stress response, whereas OT could decrease it [34, 35]. Population density of animals often fluctuates greatly in the field under the influence of climate, food, predators or parasites. High density population is often accompanied by an increase in crowding, unfamiliar encounters and shortages of food or shelter, which may act as social or physiological stressors in animals. Our recent laboratory studies have shown that high density as an environmental stressor can decrease OT expression but increase AVP expression in the amygdala (AMYG), medial preoptic area (MPOA) and PVN brain areas of male Brandt’s voles. Fighting decreases OT expression in MPOA but increases AVP expression in AMYG [14]. In additional experiments, injection of OT in brains reduced aggression of Brandt’s voles, but injection of OT and OTR antagonists increased aggression [36]. AVP in brains (ventrolateral hypothalamus, VLH; anterior hypothalamus, AH; ventromedial hypothalamus, VMH) can increase intermale aggression in golden hamsters (*Mesocricetus auratus*) [37], male prairie voles (*Microtus ochrogaster*) [38] and male Syrian hamsters (*Mesocricetus auratus*) [39]. However, AVP release patterns may be distinct between lateral septum and the bed nucleus after intermale aggression [40].

When rodents face social stress, their neuroendocrine response may occur in different brain regions. PVN is a component of the HPA axis of the stress regulation pathway and consists of many nerves that synthesize various neuropeptides (such as CRH, OT, AVP, etc.)[41]. The AMYG plays an important role in regulating behavioral or physiological responses to social stress [42]. Psychological stress can increase the expression of CRH mRNA and its content in the central amygdala cells of rats [43]. When faced with fear and aversion stress, the MeA brain area is fully activated in mice, leading to neurogenic hypertension [44, 45]. OT expressed in the MPOA is very important for mating behavior, social recognition, and parental care behavior [34, 46, 47]. AVP expression in MPOA plays an important role in aggressive behavior and territory marking [48–50].

OT and AVP play a significant role in social recognition, aggression, parental care, mating [51–56]. Many previous studies have documented that OT, AVP and their receptors (i.e., OTR and AVPR) regulates aggressive behavior in mammals. High levels of aggression behavior in hamsters were closely associated with high AVP expression [50, 57, 58]. A high level of OT expression significantly decreased aggressive behavior in female rats (*Rattus norvegicus*) [59, 60].

**Fig. 1:**
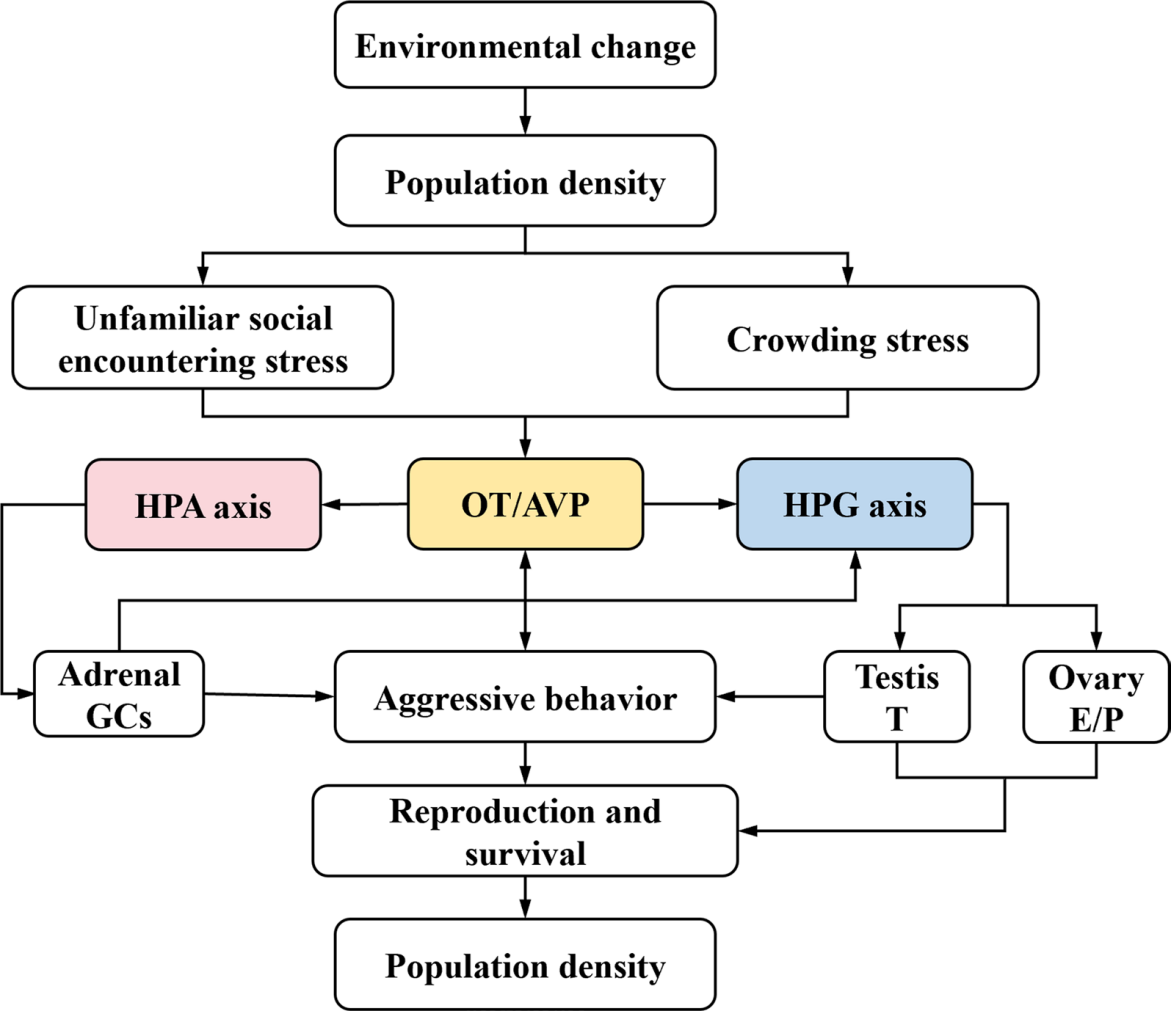
Hypothesis of density-dependent behavioral regulation (and then population regulation) through OT/AVP systems. High density would increase crowding stress due to shortage of space, and unfamiliar encountering stress due to increased interactions with strangers, which would increase the AVP expression or decrease the OT expression. High-level of AVP and low-level of OT would alter HPA and HPG, which further elevate aggressive behavior by increasing glucocorticoids (GCs, corticosterone in rodents), and inhibit reproduction by reducing release of GnRH, estrogen (E), progesterone (P), and testosterone (T). High-level of AVP and low-level of OT may increase aggressive behavior directly, and high level of aggressive behavior could reduce OT and increase AVP, and to reduce survival rate due to direct fighting

Based on the current knowledge (Fig. 1), we hypothesize that variation in population density will be reflected in variation of AVP and OT in the brains of small rodents in field populations. High population density as a stressor would increase both crowding stress and aggressive stress due to the increased encounters with unfamiliar individuals within the population, which would then increase AVP expression but decrease OT expression in specific brain regions of animals [14]. High population density induced the change of OT and AVP, further increases in aggression behavior of animals, and further increased social stress, which in return increased AVP expression but decreased OT expression. The high density induced reciprocal enhancement between OT/AVP and aggression behavior would promote the level of aggression and stressful neuro-peptides. High levels of aggression should negatively affect population growth rates due to increased mortality or reproductive failure caused by fighting or interference. High-level of AVP and low-level of OT would alter HPA and HPG axis, which further elevate aggressive behavior by increasing stressful hormone (e.g., CRH, ACTH, GCs, CORT level and inhibit reproduction by reducing release of reproductive hormone (e.g., GnRH, FSH, LH, estrogen, progesterone, testosterone).

Brandt’s voles (*Lasiopodomys brandtii*) occur in the steppe grasslands of Inner Mongolia, China, and Mongolia. Brandt’s voles are social animals, living as a family in a highly organized territory. They are polygamous during the breeding season (i.e., from May to August). Both extrinsic and intrinsic factors affect their population fluctuations [61–63]. Our previous laboratory studies on Brandt’s voles demonstrated that at high density, AVP expression increased and OT expression decreased in association with crowding stress and the observed increase of aggression behavior [14]. We now test whether these associations also occur in field conditions. We test our prediction that high density will increase AVP expression but reduce OT expression in specific brain regions, and increase the aggressive behavior of Brandt’s voles in large semi-natural field enclosures.

## Materials and methods

### Experiment 1

Since 2010, large manipulation experiments studying the population dynamics of Brandt’s voles have been conducted at the Maodeng pasture (44° 11′ N, 116° 27′ E) of Xilinhot, Inner Mongolia, China [64, 65]. We constructed 24 enclosures (each 60 × 80 m; 0.48 ha, Additional file 1: Fig. S1) with treatments of control, food supplementation (adding 100 g laboratory rodent chow pellets to each family weekly during the growing season), livestock grazing (allowing 40 adult sheep to graze for a half day or a whole day biweekly during the growing season to create light and moderate grazing pressure) and rainfall supplementation (adding extra 50 or 100 mm precipitation during the growing season, creating a light and moderate rainfall supplement; the local annual precipitation is less than 300 mm). The walls of the enclosures were built with steel plates, with 1 m deep under the ground to prevent immigration and emigration of rodents and immigration of small predators. The top of the enclosures was covered by nylon wire net to prevent avian predation.

This study was conducted in 2014. Thirteen male–female pairs of voles were released into each of the 24 enclosures as founder populations in late April 2014. The population density was surveyed monthly using live traps. By early October, population density of voles ranged from 64 to 492 voles in the various enclosures, which is comparable to the density variation observed in natural populations. Variation in population density among treatments and replicates was large, which provided an opportunity of studying the density-dependent changes in the OT/AVP systems of Brandt’s voles. Six adult male Brandt’s voles in each enclosure were sacrificed in early October for our neurobiological research. Once captured, they were immediately anesthetized with sodium pentobarbital (1 mg/10 g body mass) and terminated by decapitation. The whole brain was immediately removed from the skull to a tinfoil paper within approximately 30 s and then was put on dry ice to freeze rapidly (5 min). The fresh brain tissues were then kept in a liquid nitrogen and transported to our lab in Beijing. Because vole populations did not successfully establish in seven enclosures in 2014, only seventeen enclosures were available for statistical analysis in this study.

To study the association between aggressive behavior and population density of voles, behaviors of voles were surveyed in one high and one low population density enclosure in early October 2014. Because these observations lacked replicates, we repeated behavioral observations in 2017. Using video cameras, we repeated the study to collect behavior data in twenty-four different enclosures for a succession of 11 days in September 2018 to determine the relationship between aggression behavior and population density in field conditions. Observations were made in late afternoon (16:00–18:00). We chose randomly three families in each enclosure and recorded the behavior of voles on the ground for at least 0.5 h during each observation. We classified the observed behaviors into two types: aggression and chasing [66].

### Experiment 2

The density variation in the first enclosure experiment was created by food addition, grazing, and rainfall addition which may obscure the association between population density and OT/AVP expression.

In 2018, we conducted a density manipulation experiment at the same site using 12 of the 24 enclosures (each 60 × 80 m; Additional file 1: Fig. S1) at the Research Station of Animal Ecology on the Grassland, Xilinhot, China. As compared to experiment 1, experiment 2 aimed to examine the neurological effects of density on voles by excluding the co-varying treatments of grazing, and supplemented rainfall. Three density levels were established by introducing different numbers of voles into enclosure as founder populations (Low density: 6 male–female pairs of voles, moderate density: 12 male–female pairs of voles, and high density: 18 male–female pairs of voles). Voles used in this experiment were all captured from the field in April 2018. Vole populations grew freely in enclosure from May to October and were investigated regularly by using Capture-Mark-Recapture methods (details in Li et al*.* 2016) [64, 65]. To ensure enough food for each individual, we added 150 g rodent chow for each vole family weekly. In early October, six adult male Brandt’s voles from each density level were sacrificed for measuring the mRNA and protein expression of OT and AVP as well as their receptors. Once captured, we used the same sampling procedure described above in experiment 1.

To study the impact of population density on aggressive behavior, we recorded the behaviors of voles in these enclosures in late September 2018 by means of video cameras. Observations were made in late afternoon (16:00–18:00). We randomly chose three families in each enclosure and recorded the behaviors of voles on the ground for at least 0.5 h during each observation. We classified the observed behaviors into two types: aggression and chasing.

### Real-time PCR

Tissues of male voles were from bilateral brain regions of AMYG, MPOA and PVN from different treatments. Total RNA was extracted according to the methods by Trizol (Invitrogen, 1596-026). First-strand cDNA was synthesized using reverse transcriptase kit with Oligo (dt)18 primer (Fermentas, #K1622). The relative quantification of the OT, OTR, AVP and AVPR gene was determined using SYBR Green PCR kit (Thermo, #K0223). The data were analyzed by the 2[− ∆∆Ct] method. A standard curve was prepared by serial tenfold dilutions. Primers and probes are designed for the ot, otr, avp and avpr genes (Additional file 2: Table S1).

### Western blotting

Tissues of male voles were from bilateral brain regions of AMYG, MPOA and PVN. The tissues were sonicated in 150–250 µl homogenization buffer containing 1% sodium dodecyl sulfate (SDS). The concentration of protein was determined using the BCA Protein Assay Kit (Thermo, PIPI2323). After electrophoresis fractionation (Bio-Rad) and transferring onto a nitrocellulose membrane (Millipore, HATF00010), we blocked the membrane in skim milk or SuperBlock T20(TBS) Blocking Buffer (Thermo, 37536) for 1 h. After blocking, the membrane was then incubated with either antibodies against OT (1:1500, Abcam, ab67457), OTR (1:3000, Abcam, ab181077), AVP (1:1000, SANTA, sc-390702), AVPR (1:1000, Abcam, ab187753), or GAPDH (1:2000, CST, #5174). Finally, Blots were developed by ECL TMB Substrate Solution (Millipore, WBKLS0100) and imaged with the Tanon Infrared Imaging system (Tanon-5200).

### Statistical analyses

In the enclosure experiment, we used linear models to test the effects of population density on aggressive behavior, protein expression and mRNA expression levels for a cohort of genes, including OT, OTR, AVP and AVPR), in different brain areas, AMYG, MPOA and PVN. The OT/AVP system in the brain regions have been shown to participate in stress regulation and social behaviors such as reproduction, intimacy, aggression, parental care and social cognition [42, 49, 67–70]. We used ANOVA to test the significant effects of enclosure density on the expression levels of mRNA and relevant protein for four genes (OT, OTR, AVP and AVPR) in three brain areas. We used the Shapiro–Wilk test and Levene’s test to check the normality and homogeneity of variance assumptions. All statistical analysis was conducted in R (version 3.5.1).

## Results

### Experiment 1

#### Effects of population density on aggression behavior

**Fig. 2:**
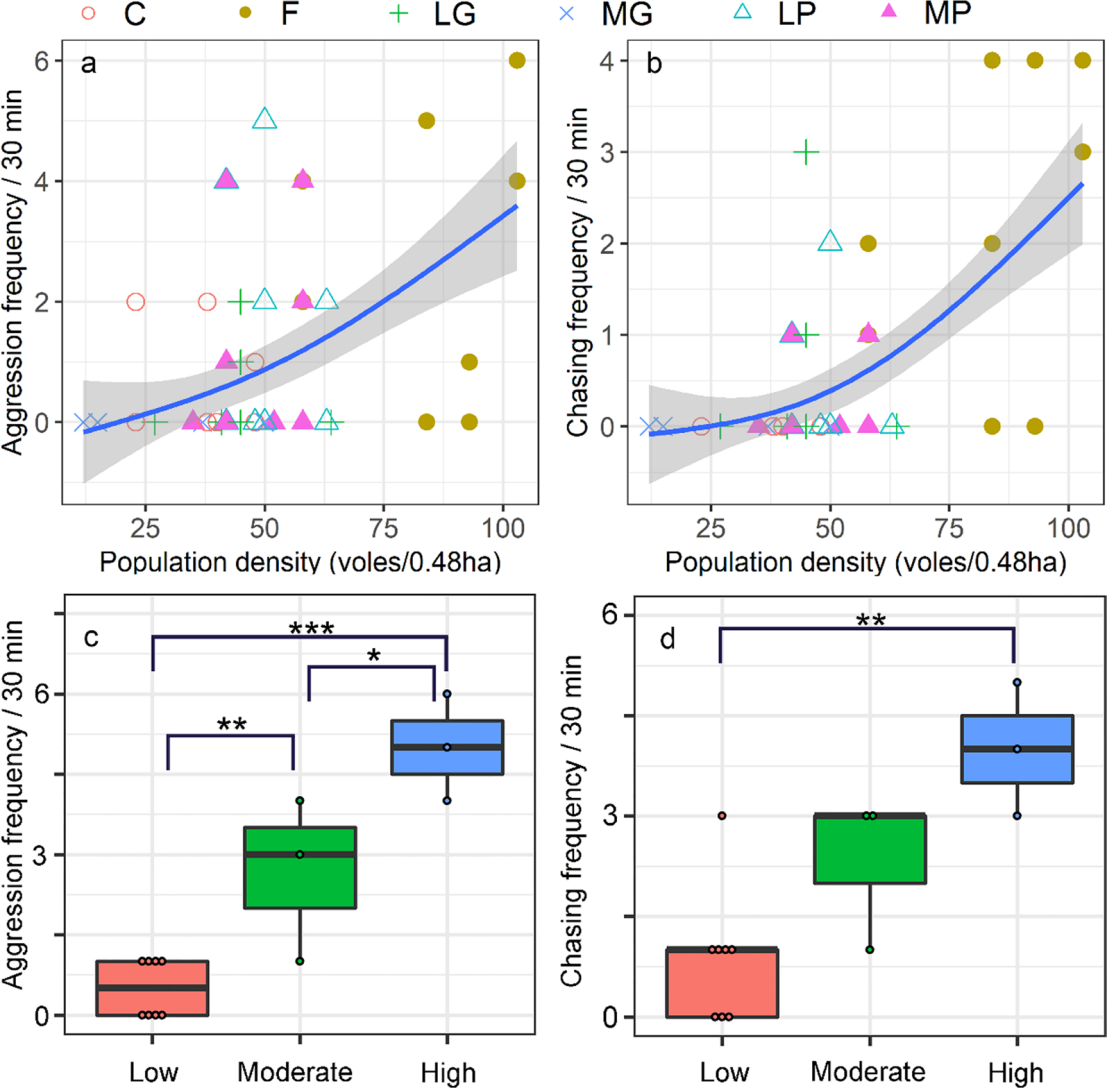
Effects of population density in field enclosures (**a**, **b**) on frequency of aggression or chasing behaviors between voles. For (**a**) and (**b**), C, control group; F, food supplementation; LG, light grazing; MG, moderate grazing; LP, light precipitation supplementation; MP, moderate precipitation supplementation. For (**c**) and (**d**), Low: low density populations; Moderate: moderate density populations; High: high density populations. Data are shown as mean ± SE. Asterisks indicate significant differences between density groups (*P < 0.05; **P < 0.01; ***P < 0.001)

In experiment 1 (2014), voles in a high-density population showed a significantly higher frequency of aggression and chasing behavior than voles in a low-density population (Additional file 1: Fig. S2). In experiment 1 (2017), aggression frequency between voles was positively associated with increased population density in 24 enclosures (F = 17.5, *P* < 0.001; Fig. 2a). The chasing frequency between voles was also positively related to population density (F = 14, *P* < 0.001; Fig. 2b).

#### Effects of population density on mRNA expression of OT/AVP

**Fig. 3:**
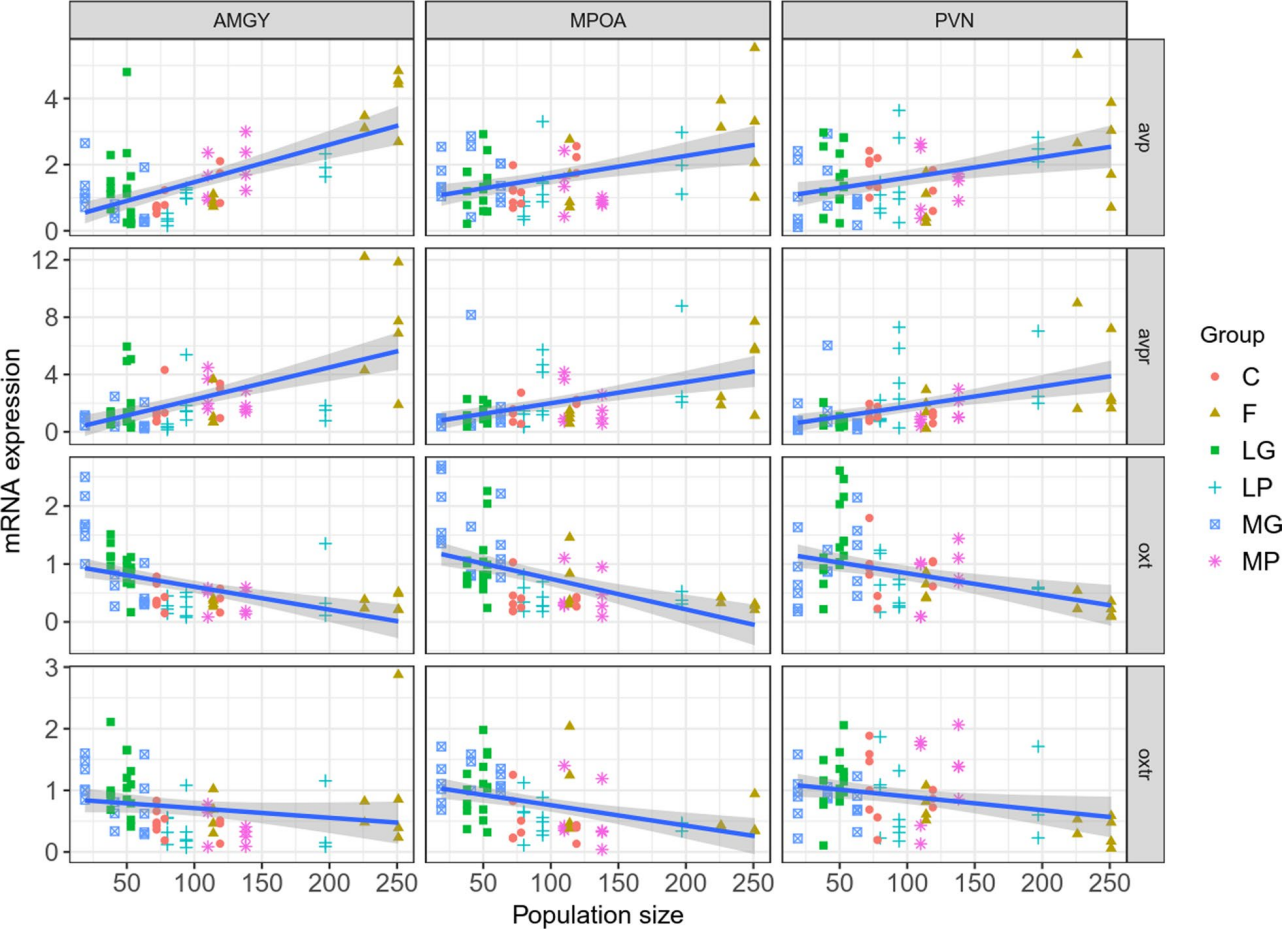
The association between population density and mRNA expression of AVP, AVPR, OT and OTR in brain areas of AMYG, MPOA and PVN. The fitted lines are presented with their 95% confidence interval. C, control enclosures; F, food supplementation enclosures; LG, light grazing enclosure; MG, moderate grazing enclosure; LP, light rain supplementation enclosure; MP, moderate rain supplementation enclosure. Data are shown as mean ± SE. Asterisks indicate significant differences between density groups (*P < 0.05; **P < 0.01; ***P < 0.001)

Population density showed significant and positive effects on mRNA expression of AVP and its receptor (AVPR) in three brain regions of voles in the enclosure (Fig. 3; For more details on statistical significance see Additional file 2: Table S2). In addition, mRNA expression of AVPR differs between the enclosure treatments in AMGY (F_5,10_ = 4.2, *P* = 0.028), livestock grazing enclosures were lower expression compared with that in control enclosures. The difference in mRNA expression of AVP/AVPR was not significant between different brain areas (all *P* > 0.05; Fig. 3).

Population density showed significant and negative effects on mRNA expression of OT and its receptor (OTR) of voles in the enclosure (OT: *P* < 0.001; OTR: *P* < 0.05), except OTR in the brain areas of AMYG (OTR: F_1,67_ = 2.38, *P* = 0.127; Fig. 3; Additional file 2: Table S2). The mRNA expression of OT shows significant difference of enclosure treatments in the MPOA (F_5,10_ = 9.2, *P* = 0.0017), OTR has the difference in AMYG and MPOA (AMYG: F_5,10_ = 4.15, *P* = 0.027; MPOA: F_5,10_ = 6.58, *P* = 0.0058, Fig, 3).

### Experiment 2

#### Effects of population density on aggression behavior

Vole population density significantly affected the aggression frequency (F_2,11_ = 29.4, *P* < 0.001; Fig. 2c) and chasing behavior between voles (F_2,11_ = 10.6, *P* = 0.003; Fig. 2d). Compared to voles in low density populations, voles in high density populations and moderate density populations exhibit significant higher aggression frequency (High VS Low: t = 4.5, *P* < 0.001; Moderate VS Low: t = 2.2, *P* = 0.01; Fig. 2c). Chasing behavior was also higher for voles in high density populations compared to that of voles in low density populations (High VS Low: t = 3.1, *P* = 0.002 Fig. 2d). No significant difference in chasing behavior was found for voles between low density population and moderate density population (Moderate VS Low: t = 1.46, *P* = 0.13; Fig. 2d).

#### Effects of population density on mRNA expression of OT/AVP

**Fig. 4:**
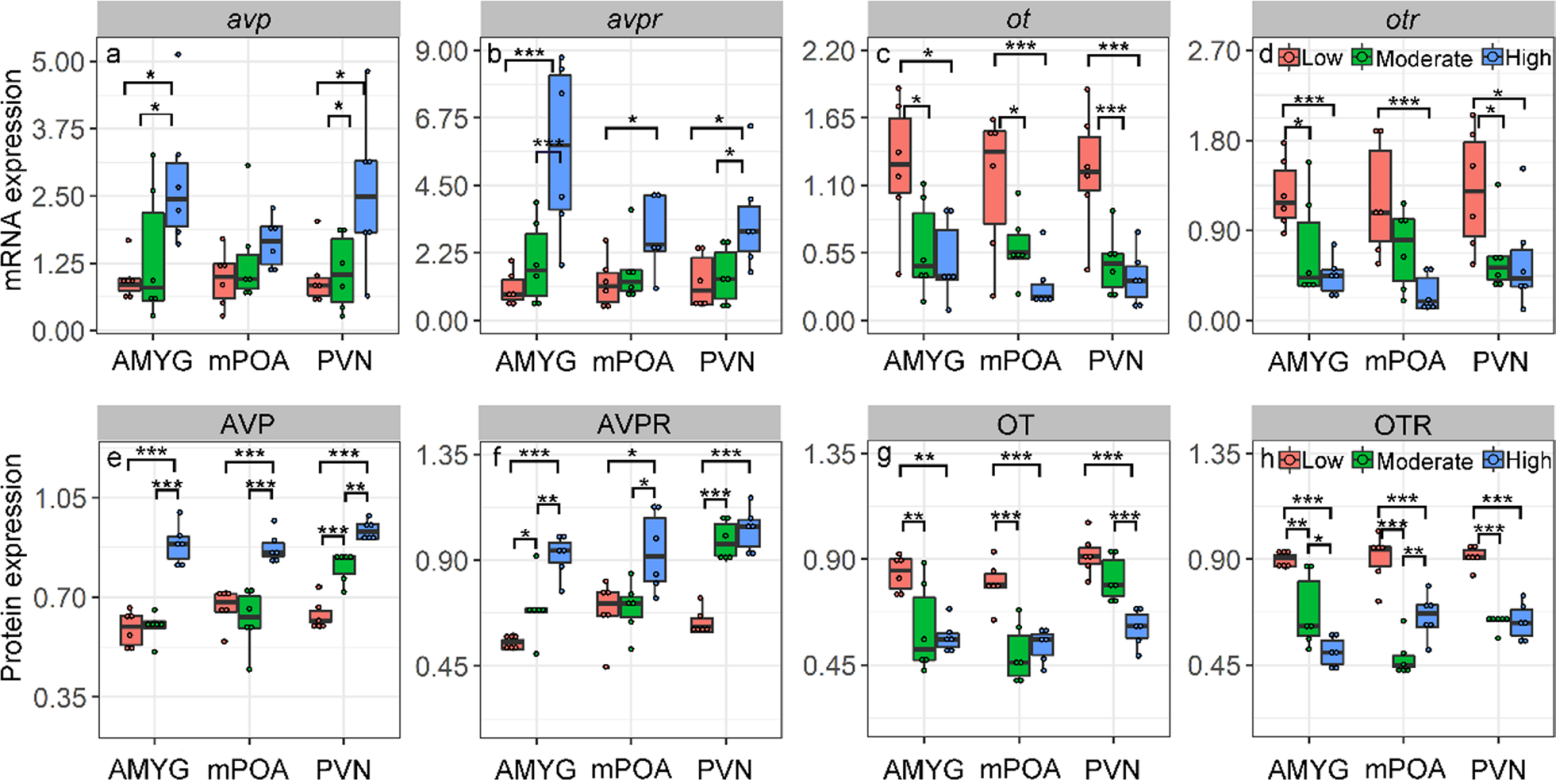
Effects of population density (low, moderate and high density) on mRNA expression of AVP/OT **a**–**d** and protein expressions of AVP/OT **e**–**h** in brain areas of AMYG, MPOA and PVN. Data are shown as mean ± SE. Asterisks indicate significant differences between density groups (*P < 0.05; **P < 0.01; ***P < 0.001)

Population density significantly affected the level of AVP and AVPR mRNA expression in AMYG (AVP: F_2,15_ = 4.9, *P* = 0.022; AVPR: F_2,15_ = 10.3, *P* = 0.0015) and PVN (AVP: F_2,15_ = 4.88, *P* = 0.023; AVPR: F_2,15_ = 4.9, *P* = 0.022). Voles in the high-density group exhibited higher levels of AVP and AVPR mRNA expression compared to those in low or moderate density groups (Fig. 4a, b). In MPOA, compared with low population density group, the level of AVPR mRNA expression was higher in high population density group (t = 2.73, *P* = 0.04). However, no difference in the AVP mRNA expression in MPOA was found between different population density groups.

We found OT and OTR RNA expressions were affected by population density in AMYG (OT: F_2,15_ = 5.7, *P* = 0.014; OTR: F_2,15_ = 7.2, *P* = 0.006), MPOA (OT: F_2,15_ = 7, *P* = 0.007; OTR: F_2,15_ = 7.45, *P* = 0.0056) and PVN (OT: F_2,15_ = 10.9, *P* = 0.001; OTR: F_2,15_ = 3.77, *P* = 0.047). Voles in moderate and high-density groups had higher OT and OTR mRNA expression than voles in low density groups (Fig. 4c, d).

#### Effects of population density on protein expressions of OT/AVP

Population density regulated the protein expressions for both AVP and AVPR of voles in AMYG, MPOA and PVN. In all tested brain areas, we found the protein expressions of AVP and AVPR were significantly higher in high population density group than that of low population density group (Fig. 4e, f). We found high population density decreased the protein expression of OT/OTR of voles (OT: F_2,45_ = 5.3, *P* = 0.008; OTR: F_2,45_ = 6.7, *P* = 0.002). In all brain areas, we found that protein expressions of OT were significantly higher in low density group than those at high density group (Fig. 4g). The protein expressions of OTR are similar with OT that were all higher in low density group than those of high population density group (Fig. 4h).

## Discussion

Although OT/AVP are well known to regulate social behavior in mammals, their roles in the density-dependent behavioral population regulation of small mammals have not been investigated in field conditions. Since 2010, in order to explore the impact of human activities and climate change on vole population density, we have set up four treatment groups of 24 large-scale field enclosures and found that the various environmental disturbances can cause a large variation in population density. This provided an opportunity to test effect of density-dependence from a neurobiological perspective. In 2014, we collected brain samples of male Brandt’s voles to explore the relationship between different population densities and the expression of OT/AVP system, and we found in support of our hypothesis that at high population densities, mRNA expression of AVP/AVPR increased, but decreased the mRNA expression of OT/OTR in the AMYG, MPOA and PVN brain regions of Brandt’s voles, which linked to the increasing aggression frequency and chasing behaviors. For eliminating the interference of the experimental treatments, the density enclosure gradient experiment was further set up in 2018 to verify the relationship between the neuropeptide OT/AVP and population density. In this study, we found a similar trend of mRNA and protein expression OT/AVP system in the specific brain regions of Brandt’s voles with the population dynamics, and the behavioral results are also consistent with 2014. Our results suggest that changes in OT/AVP expression are likely a result of increased psychosocial stress of voles experienced during overcrowding. Thus OT/AVP systems could be used as a density-dependent indicator of population changes in small mammals. However, its roles in regulating density-dependent aggressive behavior, and possibly thereby affecting the population dynamics of small rodents in field conditions need further investigation.

Our results are consistent with those in laboratory studies of the function of OT and its receptor (OTR) in regulating aggressive behavior of rodents and mammals [63, 71–74]. Increasing OT level in brains could promote the social affiliation and social bonding or reduce aggressive behaviors of animals [34, 35, 75], while decreasing OT levels would increase aggression behavior [76]. Based on experiments of injecting OT and oxytocin receptor antagonist (OTRA) into the lateral ventricle of C57mice, it is shown that OT could promote social communication between groups and increase individuals’ pro-social behaviors [77]. OT regulates the HPA axis stress response level by inducing the production of gamma-aminobutyric acid, then reduces the release of CRH and GCs, and finally reduces defensive and aggressive behaviors [34, 78]. Similarly, AVP and its receptor are important in regulating the aggression behaviors [79]. Chronic mild stress can increase the expression of AVP in the PVN area of male rats [80], and increasing of AVP or its receptor levels in special brain area could promote aggression behavior [37–39]. In our previous study, blockade of OT in the nucleus accumbens area can increase the aggressive behavior and reduce the prosocial behavior of the Brandt’s voles [36], suggesting OT could be involved in behavioral or population regulation of Brandt’s voles in field conditions.

In this study, we found that in two field experiments high population density was associated with increased AVP/AVPR expression, and decreased the OT/OTR expression, which correspond well to the increased frequency of aggression and chasing behaviors. These results are entirely consistent with our previous studies under laboratory condition, and we found that crowding stress plays an especially important role in density-dependent regulation of OT and AVP [14].In another study, we found aggression of Brandt’s voles was reduced by injection of OT but increased by injection of OT antagonists and OTR antagonists [36]. Thus, density-induced OT or AVP and their receptors are able to regulate aggression behavior which can then regulate population density. Therefore, OT/AVP systems in the rodent brain are essential for mediating density-dependent aggressive behavior and population regulation.

Many environmental stressors could increase AVP expression of animals, such as repeated restraint stress on rats, forced swimming on rats [81]. Chronic stress with high level releasing of hypothalamic CRH and peripheral GCs can cause depressive symptoms in rats and humans [82]. AVP was also observed as a major factor to regulate depression, especially in two hypothalamic structures SON and PVN which produced plasma vasopressin [21]. The administration of OT can prevent stress-induced anxiety, but this is dose-dependent [83]. In high density population, voles exhibited low level of OT, which can’t alleviate crowding-induced anxiety, resulting in low fitness of individuals and population collapse. High population density could act as an environmental stressor (e.g., crowding or aggression, food or space shortage). Our previous study indicated that high housing density could decrease OT or its receptor but increase AVP of its receptors [14], but it is not clear whether such associations operate in all field conditions. In animal experiments, the behavioral effects of OT are ‘prosocial’, including physical contacts, inter-individual interactions, increased trust, diminished fear and anxiety behavior, increased risk-taking behavior [70]. Furthermore, OT system involves in the social preference in male rats and mice, social avoidance can also be reversed by OT in amygdala [84]. High population density increases the frequency of social behavior activities and contacts among the animals; however, the subsequent long-term crowding and aggressive stress are even more harmful to the individuals. In this study, we showed that high population density increased AVP/AVPR expression but decreased the OT/OTR expression, and aggression behavior of voles was also increased in semi-natural conditions. Based on manipulation experiments of OT or its receptor of voles [36] as well as many previous studies on rodents (see above), the high population density-induced decrease of OT or increase of AVP could promote aggressive behavior of voles. Based on our fighting experiments of Brandt’s voles in the laboratory, aggression behavior as a stressor would further decrease OT expression in MPOA, suppressing social recognition and parental care behavior [69], but increase AVP expression in AMYG, increasing defense-aggressive behavior and suppressing cognitive emotion-regulation, which is a fundamental skill for normal social interaction [14, 85]. The chronic crowding cognitive and psychological stress, decrease of OT or increase of AVP could promote aggressive behavior in voles. The reciprocal facilitation between OT/AVP expression and aggressive behavior could be an important component of population regulation in small rodents (Fig. 1). Food in the natural environment is limited and can act as a key factor to control animal populations. Free competition among individuals for food would ultimately exhaust resources and result in habitat destruction and mass starvation. There are many studies indicating that the frequency and intensity of aggression are positively associated with population density [86]. In our study, we found that the frequency of aggression behavior of voles is positively associated with the population density in field enclosures, supporting our prediction. We also revealed that high population density-related crowding and fighting were associated with increased AVP/AVPR expression but decreased OT/OTR expression, and high population density increased frequency of aggressive behaviors [87].

The HPA, HPG and AVP/OT systems are closely related to each other in response to change of population density. Some physiological indicators such as circulating CORT levels, adrenal gland size, and reproductive organ size have been used to index the level of density-dependent stress on population of animals [7, 88–91]. Under high density condition, high level of hypothalamic CRH and peripheral GCs release can cause depressive symptoms in rats and humans [82], and would have an inhibitory feedback on the HPA stress axis [15, 16], and then on the reproductive axis. The high levels of CRH and GCs can reduce GnRH, LH, FSH and the reproductivity of animals [22, 92]. A recent study reveals that GnRH expression in brains is closely associated with population density of wild meadow voles (*Microtus pennsylvanicus*) using large-scale field enclosures [93]. OT antagonists or AVP injected in the cerebral ventricles had the same inhibitory effects on female sexual behavior in rats [94, 95]. OT can inhibit the activity level of the MeA and reduce the connectivity between the AMYG and the autonomic nervous system (responsible for fear responses), while the function of the parasympathetic nervous system is enhanced, reducing the release of ACTH and GCs, thereby reducing the individual's level of fear and anxiety and decreasing the aggressive behavior of animals [30, 96]. However, AVP could promote release of CRH, ACTH and GCs [14, 16], Because OT/AVP expression in the brain is strongly correlated with population density of voles, we recommend that they can be used as a fundamental indicator reflecting population changes or density-dependence stress of small rodents.

Although injection of OT or its or OTR antagonists in brains could alter aggression of Brandt’s voles under laboratory condition [36], the effects of AVP and its receptor in regulating behavior of voles have not been manipulated. The effects of OT/AVP on HPA/HPG axis regulation in Brandt’s voles are still unknown. Thus, the roles of OT/AVP system in regulating behavior or and populations of Brandt’s voles under field conditions need further investigation.

## Conclusion

Density-dependency is an important phenomenon in regulation of animal populations; however, its neurobiological mechanism remains unclear. In this study, by using large-scale field enclosures, we firstly demonstrated that the mRNA and protein expression of OT and AVP in specific brain regions are significantly associated with crowding and aggressive stress under changing population density of Brandt’s voles.

Our results indicate that density-dependent changes of OT/AVP systems are likely a result of increased psychosocial stress of Brandt’s voles experienced during overcrowding, and they can be used as an indicator reflecting density-dependent stress that results in population changes of small rodents.

## Supplementary Information

**Additional file 1. Table S1.** Sequences of the primers for qPCR experiments in this study.Table S2. Linear mixed model results on the relationship between population density and expression of some genes in AMYG, MPOA and PVN.

**Additional file 2. Fig. S1.** Experimental enclosures in the research station of Inner Mongolia grassland (photo by Guoliang Li). **Fig. S2.** Behavioral observation in high- and low-density field enclosures in 2014. (a) Difference in chasing frequency of voles between high-density and low-density enclosures. (b) Difference in chasing frequency per individual vole between high-density and low-density enclosures.

## Data Availability

The datasets used and/or analyzed during the current study are available from the corresponding author on reasonable request.
