# Supplementary material for: Population variation alters aggression-associated oxytocin and vasopressin expressions in brains of Brandt’s voles in field conditions
Source: Front Zool. 2021 Oct 30;18:56. doi: 10.1186/s12983-021-00441-w (PMC8557550; doi:10.1186/s12983-021-00441-w)
Supplement: Supplementary file 1 — Additional file 1. Table S1. Sequences of the primers for qPCR experiments in this study.Table S2. Linear mixed model results on the relationship between population density and expression of some genes in AMYG, MPOA and PVN. [file 12983_2021_441_MOESM1_ESM.doc]

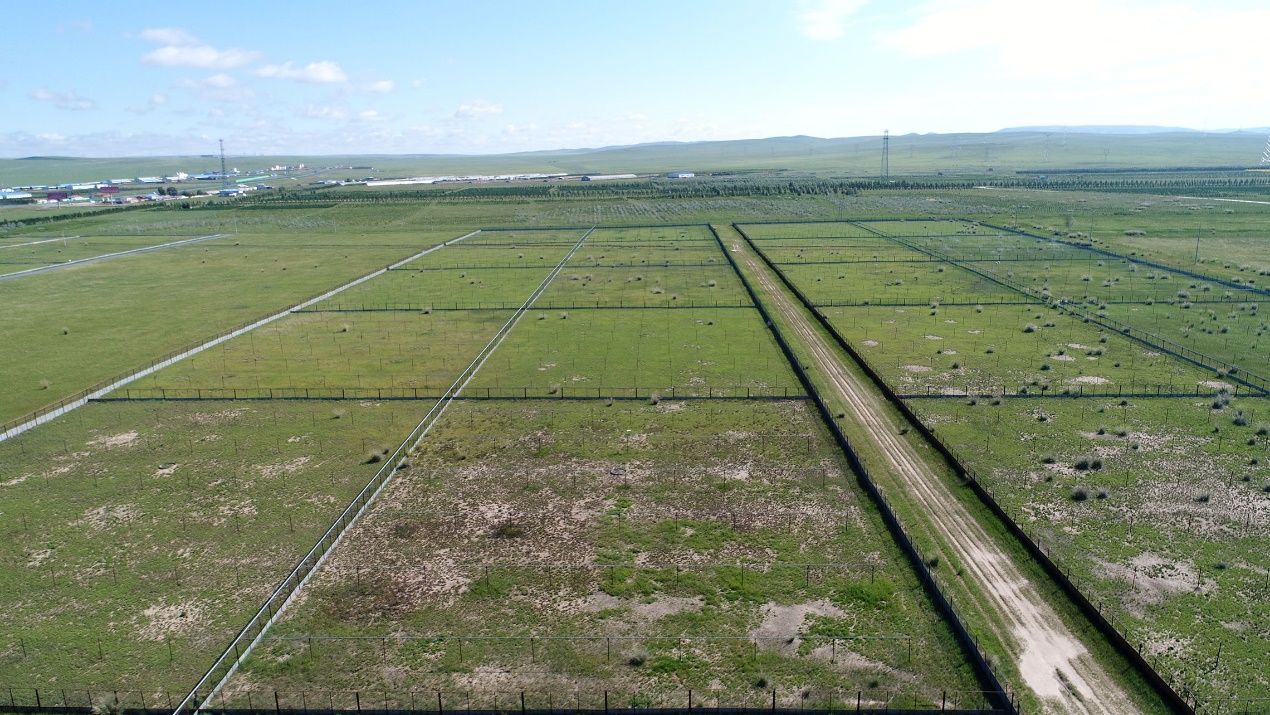


**Fig. S1**. Enclosures in the Inner Mongolia grassland (photo by Guoliang Li)


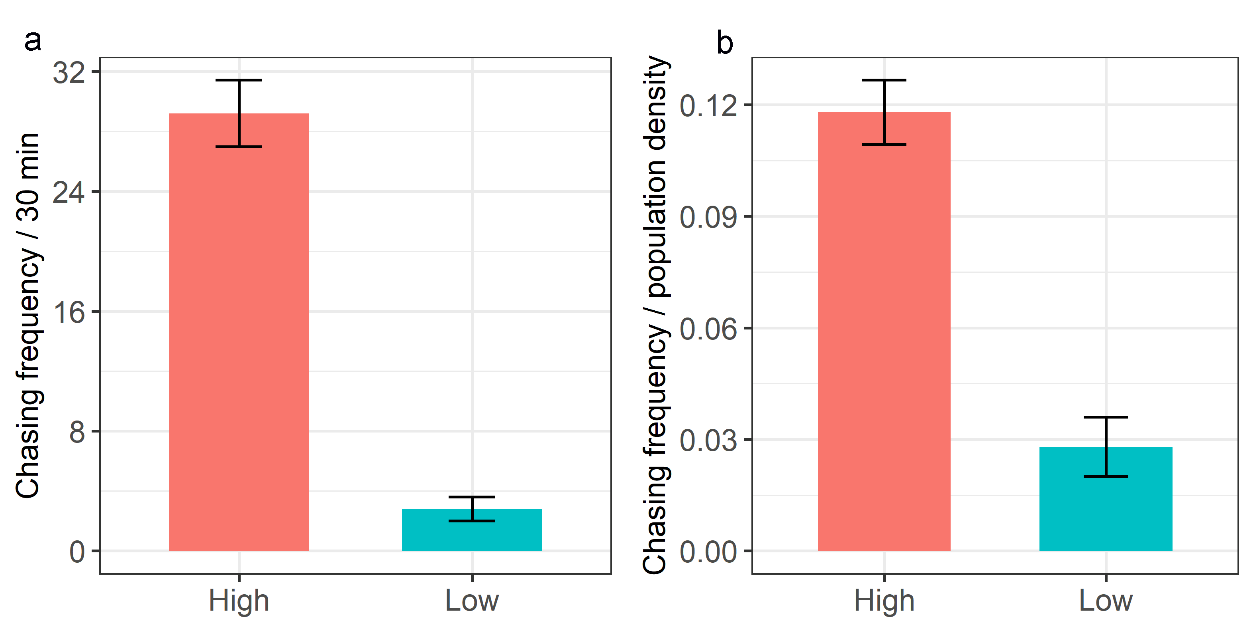
 **Fig. S2**. Behavioral observation in high- and low-density field enclosures in 2014. (a) Difference in chasing frequency between voles from high-density and low-density enclosures. (b) Difference in chasing frequency per individual between high-density and low-density enclosures.
