# Supplementary material for: Population variation alters aggression-associated oxytocin and vasopressin expressions in brains of Brandt’s voles in field conditions
Source: Front Zool. 2021 Oct 30;18:56. doi: 10.1186/s12983-021-00441-w (PMC8557550; doi:10.1186/s12983-021-00441-w)
Supplement: Supplementary file 2 — Additional file 2. Fig. S1. Experimental enclosures in the research station of Inner Mongolia grassland (photo by Guoliang Li). Fig. S2. Behavioral observation in high- and low-density field enclosures in 2014. (a) Difference in chasing frequency of voles between high-density and low-density enclosures. (b) Difference in chasing frequency per individual vole between high-density and low-density enclosures. [file 12983_2021_441_MOESM2_ESM.doc]

**Table S1.** Sequences of the primers for qPCR experiments in this study.

| Gene | Primers | Sequence(5'- 3') |
| --- | --- | --- |
| Ot | Forward | TGCCAGGAGGAGAACTACC |
|  | Reverse | TCCGAGAAGGCAGACTCAG |
| Otr | Forward | CTCCCACCTATTTCTACTACC |
|  | Reverse | TCATTTCCCACTCCTTGTC |
| Avp | Forward | ACGCTCTCCGCTTGTTTC |
|  | Reverse | ACGCTCTCCGCTTGTTTC |
| V1aR | Forward | ATGGCACCAAAGCCCAAG |
|  | Reverse | ACAAGGCGTGACCAGAAG |
| Gapdh | Forward | ATCACTGCCACCCAGAAG |
|  | Reverse | TCCACGACGGACACATTG |

| **Table S2** The linear mixed model results on the relationship between population density and expression of some genes in AMYG, MPOA and PVN. | | | | |  |
| --- | --- | --- | --- | --- | --- |
|  |  |  |  |  |  |
| Brain area | Gene | F value | P value | Correlation coefficients |  |
| AMGY | *avp* | 15.6 | <0.0001 | 0.63 |  |
|  | *avpr* | 7.57 | <0.0001 | 0.58 |  |
|  | *ot* | 0.05 | <0.0001 | -0.49 |  |
|  | *otr* | 0.11 | 0.1272 | -0.19 |  |
| MPOA | *avp* | 11.6 | <0.001 | 0.42 |  |
|  | *avpr* | 10.4 | <0.0001 | 0.49 |  |
|  | *ot* | 2.20 | <0.0001 | -0.53 |  |
|  | *otr* | 3.75 | 0.0002 | -0.42 |  |
| PVN | *avp* | 5.89 | 0.0017 | 0.37 |  |
|  | *avpr* | 3.86 | <0.0001 | 0.47 |  |
|  | *ot* | 0.003 | <0.001 | -0.40 |  |
|  | *otr* | 0.63 | 0.0263 | -0.27 |  |
